# Perceived Novelty Support and Psychological Needs Satisfaction in Physical Education

**DOI:** 10.3390/ijerph17114169

**Published:** 2020-06-11

**Authors:** Sebastián Fierro-Suero, Bartolomé J. Almagro, Pedro Sáenz-López, José Carmona-Márquez

**Affiliations:** Psychology and Sport Sciences, Faculty of Education, Universidad de Huelva, Avda. Tres de marzo s/n, 21071 Huelva, Spain; fierro.suero@ddi.uhu.es (S.F.-S.); psaenz@uhu.es (P.S.-L.); carmona@uhu.es (J.C.-M.)

**Keywords:** self-determination, secondary education, motivation, novelty support, social factors, validation, psychometric properties

## Abstract

In recent years, novelty has been proposed as a potential fourth basic psychological need. In the present study, the behavior of novelty resulting from support from the Physical Education teacher was evaluated in 723 students with an average age of 13.30 years old. The first objective was to validate the Support for Basic Psychological Needs-4 (SBPN-4) in Physical Education questionnaire, which included support for the novelty factor. The second objective was to test the mediation model in order to confirm the effect of support for novelty in relation to basic psychological needs and intrinsic motivation. The results obtained show that the Support for Basic Psychological Needs-4 (SBPN-4) questionnaire is a valid and reliable tool. On the other hand, support for novelty predicts satisfaction of basic psychological needs, particularly novelty satisfaction, which in turn predicts intrinsic motivation. These results show how the students are capable of perceiving the teacher’s support for novelty and how this positively influences their intrinsic motivation. Further investigations are required to continue developing our knowledge of the role of novelty as a basic psychological need.

## 1. Introduction

Improvement of the educational system and, moreover, the development of students happens when we increase the motivation of those involved [1]. One of the principal theories of motivation is the Self-Determination Theory (SDT) [1,2], which explains the extent to which people act according to their own will; in other words, through self-determined behavior. Particularly, it references how social and contextual factors support or hinder people’s success through satisfaction of their basic psychological needs (BPNs), such as autonomy, competence, and relatedness [2]. The aforementioned theory establishes that we all possess these innate and universal needs, whose satisfaction is essential for psychological growth, as well as optimal performance and wellbeing. In other words, experiences which involve and satisfy the BPNs generate positive emotions and foster psychological wellbeing [3]. In this sense, social surroundings can be evaluated by the degree to which they support or frustrate these BPNs. 

### 1.1. Support of Basic Psychological Needs

Amongst the social factors presented within the educational field, the role of the teacher has been shown as the most determinant [4]. In this context, support for autonomy refers to the possibility afforded to students to make choices themselves and assume responsibility whenever possible, in contrast to more controlling styles of teaching. Support for competence refers to developing effectiveness through positive feedback as opposed to more challenging or discouraging styles of teaching. Support for social relatedness consists of developing supportive and authentic participation with others rather than more impersonal or rejectionist styles of teaching [1]. Thus, the teacher can condition the behavior of their students by utilizing these strategies [5,6,7], achieving development through more or less self-determined means [4,7,8], and creating an environment that generates a degree of motivation in their students [9]. More controlling styles that do not support satisfaction of the BPNs have shown less significant learning in students and greater behavioral problems. On the other hand, school environments that support autonomy (a concept understood more broadly as the support of all BPNs) foster a lasting quality of learning, greater enjoyment towards it, or greater participation [1,4,6,7,10]. In short, teachers have a fundamental role in the development of motivation through the creation and maintenance of an optimal social and psychological environment [11].

Particularly in Physical Education, the importance of SDT has been widely studied and has yielded numerous benefits [12,13,14,15]. Previous studies have shown how perceived support for autonomy in the teacher positively predicts satisfaction of the BPNs, which in turn predicts more self-determined motivations [7,13,16]. For this reason, the role of the teacher is very important when it comes to promoting a learning context that is characterized by setting tasks and activities with adequate guidelines [17,18].

Given the importance of evaluating social factors related to the teacher from multiple perspectives, the Questionnaire on Support of the Basic Psychological Needs in Physical Education (CANPB) [19] was designed to include the roles played by all three BPNs. Thus, it does not uniquely assess support for autonomy, as instruments up to this point have done [19]. With said aim in mind, a scale has been specifically designed to assess the students’ perception of the resources that the teacher directs to the promotion of the BPNs in Physical Education (PE).

### 1.2. Novelty as a BPN

Recent studies [20] have proposed the possibility of including novelty as an additional BPN within the SDT paradigm [1,2]. Novelty has been defined as the need to experience something that has not been experienced before or that differs from a person’s daily routine [20]. Previous studies around the topic [20,21,22] have made theoretical revisions of the literature from the perspective of classical theories as well as from more contemporary foci, gathering conclusions to suggest that the study of novelty as a BPN would be valuable for further understanding. Investigational work completed until now [20,21,22,23] has produced significant results in relation to the criteria to identify a new basic psychological need, established by Ryan and Deci [1]. Thus, it has been shown that the satisfaction of novelty is related to improvements in health and psychological wellbeing, as well as to intrinsic motivation. Moreover, novelty has functioned in synergy with autonomy, competence, and relatedness and has been shown to operate in the same manner in different contexts (PE, general life, adults, adolescents, etc.). These findings show how novelty seems to behave similarly to the other BPNs. In recent years, more studies have arisen that address novelty as a fourth BPN, especially in the field of physical exercise and physical education (e.g., [23,24,25]). Novelty, as a need candidate, has passed an initial test, however, this remains a very recent line of inquiry that must be investigated further and more deeply until we are able to confirm novelty as the fourth pillar in the BPN theory [26]. 

Departing from the theoretical framework developed in the aforementioned studies on the role of novelty [20,21,22,23], teacher support for novelty could involve proposing new activities, developing methods that foster novel sensations in students, creating new learning contexts for example. Variety and novelty, despite being related concepts, are not identical [21], although something novel is probably varied. However, variation does not necessarily signify novelty, because the pupil may already be familiar with the variation and, therefore, may not perceive it as a novelty. A similar relationship exists between novelty and surprise, concepts that have been used in similar ways to each other despite being different [27]. Novelty can cause the emotion of surprise, but surprise does not necessarily imply novelty. This is because surprise comes from the discrepancy between the expectation that something will happen and it actually happening; that is, as a result of finding something unexpected [27]. When something is unexpected, it may not have been previously experienced or existed in the daily routine. However, it may indeed have been previously experienced, but the expectation that it would happen at that precise moment was very low, which entails high surprise but low novelty.

### 1.3. Present Study

The present investigation attempts to gain further knowledge on how novelty may function as a fourth basic psychological need. Particularly, following recommendations from previous studies [20,21,26], novelty is examined from the perspective of related social factors surrounding the physical education teacher, as established by SDT [2]. Currently, the focus is almost exclusively on experience one’s own novelty; so far, little conceptual work has been done on socialization practices that could characterize an interpersonal style that supports or undermines innovation [26]. On this theme, a recent publication [23] has shown how a task-involving climate positively predicts the need for novelty from the hierarchical model [28]. This study attempts to evaluate the relationship between social factors, specifically from the perspective of teacher support for BPNs, and the satisfaction of the BPNs (including novelty) amongst students. As such, a “novelty” dimension was included in the CANPB [19] and was then validated afterwards. Consequently, it impacted the third, fourth and fifth criteria of the six established by Ryan and Deci for the inclusion of new BPNs [1]. The third criterion refers to how the suggested need, in this case novelty, is essential in explaining and interpreting the empirical phenomena; in other words, it must act as a constant mediator between social and personal factors, and people’s motivation. The fourth criterion refers to how the candidate need must not be operable only when there is a deficit or thwarting of other needs. That is to say, novelty must be consistent with the idea of a growing need rather than a deficit need. The fifth criterion refers to how the proposed need must be within the appropriate category of variables, and so, in the case of novelty, it must be a predictive factor of motivation and wellbeing, not merely a consequence.

Overall, the principal objective of the study was to provide evidence for the possible inclusion of novelty as the fourth basic psychological need. With this aim in mind, two further principal objectives were formulated, the first being to adapt the Support for Basic Psychological Needs-4 (BPNS4) in Physical Education questionnaire to include the novelty factor. The second objective consisted of testing a mediation model that included support for novelty, the other BPNs and intrinsic motivation in PE. Thus, the following hypotheses were presented: (1) the BPNS4 will present adequate psychometric properties; (2) support for novelty will predict the satisfaction of BPNs (including the satisfaction of novelty), which, in turn, will predict intrinsic motivation. It is hoped that the findings obtained in this study continue to help clarify the role played by novelty as the fourth BPN contributing to the third, fourth, and fifth criteria established by Deci and Ryan [1].

## 2. Methods

### 2.1. Participants

Participants were 723 (349 boys and 374 girls) secondary school students aged between 11 and 16 years (M = 13.30, SD = 1.20). Children were recruited from one subsidized and three public secondary schools in the province of Huelva (Spain). Students from the four age groups of lower secondary education were included: 212 from Year 7, 226 Year 8, 224 from Year 9, and 61 from Year 10 (UK equivalent written). 

### 2.2. Measurement

#### 2.2.1. Support for Basic Psychological Needs-4 (SBPN4)

The SBPN4 was composed of the 12 items of the Support for Basic Psychological Needs in Physical Education questionnaire [19] augmented with four items specifically designed for this study to measure support for novelty needs. The novelty items were selected from an initial pool of 20 items. Each member of a panel of five subject-matter experts was asked to develop four items and rate the content relevance and clarity of each of the 16 items developed by the other experts. The four items with higher content validity were retained for the final version of the SBPN4 (see Appendix A).

The stem statement “In my Physical Education class, the teacher…” preceded the items of the SBPN4. The SBPN4 is comprised of four subscales: SBPN4-Autonomy (e.g., “He/She often asks about the activities to be carried out”), SBPN4-Competence (e.g., “He/She comes up with activities tailored to our skills”), SBPN4-Relatedness (e.g., “He/She helps us to resolve conflicts amicably”), and SBPN4-Novelty (e.g., “He/She frequently offers new activities”). Participants were asked to rate each item on a five-point Likert-type scale, ranging from 1 “strongly disagree” to 5 “strongly agree”.

#### 2.2.2. Basic Psychological Needs Satisfaction-4 (BPNS4)

The BPNS4 is made up of the 12 items of the Basic Psychological Needs in Exercise Scale [29,30] and the five items of the Novelty Need Satisfaction Scale [20,22]. The 17-item BPNS4 is intended to measure the satisfaction of four basic psychological needs: autonomy, competence, relatedness, and novelty. Items are preceded by the stem “In my Physical Education class…”. Exemplary items are “I feel very strongly that I have the opportunity to make choices with respect to the way I exercise” (autonomy), “I feel that exercise is an activity in which I do very well” (competence), “I feel very much at ease with the other exercise participants” (relatedness), and “I frequently feel there are novelties for me” (novelty). Respondents rated each item on a five-point Likert scale, from 1 “strongly disagree” to 5 “strongly agree”.

#### 2.2.3. Intrinsic Motivation

The intrinsic motivation subscale of a revised version of the Perceived Locus of Causality Scale [31,32] was used to measure intrinsic motivation in the Physical Education context. The intrinsic motivation subscale consists of four items (e.g., “Because Physical Education is fun”) introduced by the statement “I take part in Physical Education …”. Each item was rated on a seven-point Likert scale, from 1 “strongly disagree” to 7 “strongly agree”.

### 2.3. Procedure

The current study was conducted in accordance with the ethical principles of the American Psychological Association [33]. The study was approved by the Andalusian Ethics Committee of Biomedical Research.

Participation in the study was solicited through direct contact with school administrators and school boards. Four schools agreed to participate. As requested by the Ethics Committee, all participants provided written consent and their parents or legal guardians also gave consent for their children to participate in the study. Before administering the questionnaire to the entire sample, a pilot sample of 50 students completed the questionnaire. Our request for comments on the clarity of the questionnaire resulted in positive comments that supported the intelligibility of the items. 

The questionnaire was administered in the classroom setting. In order to ensure the anonymity and confidentiality of the responses, no identifying information was used. A member of the research team was present during the administration of the questionnaire. Completion of the questionnaire was straightforward and took approximately 20 min.

### 2.4. Data Analysis

Confirmatory factor analysis (CFA) was used to provide validity evidence of the four-factor structure of the SBPN4. The fit of the hypothesized four-factor model was tested against two competing models: a one-factor model and a second-order factor model. As there was indication of multivariate non-normality in the data (standardized Mardia’s kurtosis statistics was 37.22), a robust maximum likelihood estimation method was used [34]. Accordingly, we used the Satorra–Bentler chi-square (S-B χ2); the Satorra–Bentler scaled Comparative Fit Index (CFI), Tucker Lewis Index (TLI), and Root Mean Square Error of Approximation (RMSEA); and the Standardized Root Mean Square Residual (SRMR) to evaluate model fit. The adequacy of model fit was judged following the guidelines proposed by Hu and Bentler [35]: CFI ≥ 0.95, TLI ≥ 0.95, RMSEA ≤ 0.06 and SRMR ≤ 0.08 were considered indicative of good model fit. The fit of tested models was compared by computing the scaled difference chi-square test [36] and the difference in CFIs and TLIs. Given the sensitivity of chi square tests to sample size, a *p* < 0.01 significance level was adopted when comparing competing models.

Multi-group CFA was used to test the measurement invariance of the SBPN4 across gender. The level of measurement invariance was determined by imposing increasingly restrictive constraints to the baseline unconstrained multi-group model and comparing the fit of successive models. Following Meredith [37], configural (unconstrained), metric/weak (equal factor loadings), scalar/strong (equal factor loadings and item intercepts) and residual/strict (equal factor loadings, item intercepts and item residuals) invariance models were tested. In addition to the significance of the scaled difference chi square tests, we also considered changes in CFI equal or less than 0.010 and changes in RMSEA equal or less than 0.015 as indicative of measurement invariance [38].

A multiple mediation model was used to test if the effects of teacher support for novelty on intrinsic motivation was mediated by the satisfaction of basic psychological needs. Bootstrapped estimates of the direct and indirect effects of novelty support were computed using path analysis.

Lavaan 0.6–5 [39] and Semtools 0.5–2 [40] R packages were used to estimate CFAs and path analysis models. Descriptive statistics were computed using Jasp 0.11.1 [41].

## 3. Results

### 3.1. Factor Structure of the Support for Basic Psychological Needs-4 (SBPN4) Questionnaire

The hypothesized factor structure of the SBPN4 is a four-factor simple structure in which each item only loads on its respective factor, allowing correlations between factors but not correlations between items’ residual error terms. A CFA model was fit to the data to test this hypothesized factor structure of the SBPN4. The results of the analysis show a good fit of the four-factor model (CFI = 0.976, TLI = 0.971, RMSEA = 0.042, SRMR = 0.027). The estimates of the factor loadings and factor covariances are presented in the Table 1. Standardized factor loadings were in the range of 0.60 to 0.85 and factor correlations ranged from 0.79 to 0.89.

Given that the factors were highly correlated, possibly indicating the existence of a common factor, two competing models were also tested: a one-factor model in which all the items were forced to load on a single factor and a second-order factor model with a general factor accounting for the correlations among first-order factors. Summary fit indices from the CFAs of these models are displayed in Table 2. The second-order factor model (CFI = 0.973, TLI = 0.968, RMSEA = 0.044, SRMR = 0.030) provided a close fit to the data. The one-factor model, on the other hand, displayed only an acceptable fit to the data (CFI = 0.916, TLI = 0.903, RMSEA = 0.076, SRMR = 0.043). Nevertheless, scaled difference chi-square tests showed that the four-factor model had a better fit than both the second-order factor model (ΔS-B χ2 = 17.49, Δdf = 2, *p* < 0.001) and the one-factor model (ΔS-B χ2 = 268.36, Δdf =6, *p* < 0.001). 

### 3.2. Measurement Invariance of the SBPN4 across Gender

Measurement invariance of the SBPN4 across gender was evaluated using multi-group CFA. The measurement invariance of the four-factor model across boys and girls was examined by testing four increasingly restrictive models: (1) a model in which the same four-factor structure was estimated simultaneously in both gender groups (configural invariance model); (2) a model that extends the configural model by imposing equality constraints on factor loadings across gender (metric invariance model); (3) a model with factor loadings and item intercepts constrained to be equal across boys and girls (scalar invariance model); and (4) a model with factor loadings, item intercepts and residual variances constrained to equality across gender (uniqueness invariance model). Summary fit statistics are presented in Table 3. The configural invariance model showed a good fit to the data (CFI = 0.972, TLI = 0.966, RMSEA = 0.045, SRMR = 0.033), indicating that at least configural invariance was supported. Furthermore, when the differences in fit between adjacent models were examined, the non-significant scaled chi-square tests (*p* > 0.01 for all tests) and the small changes in CFI and RMSEA suggested that metric, scalar, and uniqueness invariance are also tenable (see Table 3). Given that scalar invariance was supported, a test of differences in latent means was conducted. We found no significant decrement in fit when equality constraints in latent means were imposed in the scalar invariance model (ΔS-B χ2 = 1.04, Δdf =4, *p* = 0.904), which provides good support for the invariance of latent means across gender.

### 3.3. Reliability, Temporal Stability, and Descriptive Statistics of the Study Variables

The internal consistency estimates (Cronbach’s α and McDonald’s ω), means, standard deviations, and correlations of SBPN4, BPNS4, and intrinsic motivation scores are presented in Table 4. The SBPN4, BPNS4, and intrinsic motivation scores showed acceptable levels of internal consistency reliability in the present study, with values of Cronbach’s α and McDonald’s ω greater than 0.70 for all the subscales. A sample of 50 students was used to measure the temporal stability with a 30-day difference between the two measurements.

Repeated measurements ANOVA of the SBPN4 scores, F (3,2166) = 510.07, *p* < 0.001, revealed that scores on SBPN4-Novelty were significantly higher than SBPN4-Autonomy (t = 17.72, *p* < 0.001, d = 0.66) and significantly lower than SBPN4-Competence (t = 15.21, *p* < 0.001, d = 0.57) and SBPN4-Relatedness (t = 15.10, *p* < 0.001, d = 0.56). The same pattern of results was observed for BPNS4 scores.

The Pearson correlation coefficients between SBPN4-Novelty and the other study variables ranged from 0.25 to 0.76. This was strongly correlated with the other SBPN4 subscales, weakly to strongly correlated with the BPNS4 subscales and strongly correlated with intrinsic motivation (60). Moreover, intrinsic motivation was moderately to highly correlated with all the SBPN4 and BPNS4 subscales, with correlations ranging from 0.40 for BPNS4-Satisfaction to 0.66 for BPNS4-Novelty.

### 3.4. Mediation Analysis

**Figure 1:**
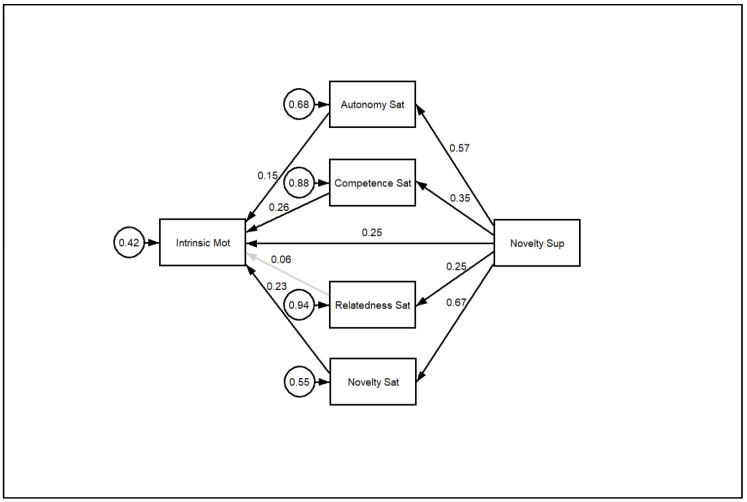
Multiple mediation model. Path analysis with standardized estimates. All estimates were significant (*p* < 0.01), except for the path from relatedness satisfaction to intrinsic motivation.

A multiple mediation analysis was performed to test if the effects of teacher support for novelty on intrinsic motivation was mediated by the satisfaction of the basic psychological needs of autonomy, competence, relatedness, and novelty. Standardized estimates of the bootstrapped path analysis are presented in Figure 1.

Bootstrap tests showed that all the paths, except for the path from relatedness satisfaction to intrinsic motivation, were statistically significant (*p* < 0.01). The multiple mediation model explained 57.6% of the variance in intrinsic motivation. On the other hand, novelty support explained 44.7%, 32.4%, 12.2%, and 6.5% of the variances of novelty satisfaction, autonomy satisfaction, competence satisfaction, and relatedness satisfaction, respectively.

The effect of novelty support on intrinsic motivation was only partially mediated by the satisfaction of psychological needs (total indirect effect: β = 0.35, *p* < 0.01; direct effect: β = 0.25, *p* < 0.01). An examination of specific indirect effects indicated that novelty satisfaction (β = 0.15, *p* < 0.01), autonomy satisfaction (β = 0.09, *p* < 0.01) and competence satisfaction (β = 0.09, *p* < 0.01) were significant mediators of the relationship between novelty satisfaction and intrinsic motivation.

## 4. Discussion

The objective of this study was to confirm how novelty behaves as a possible fourth need in the Theory of Basic Psychological Needs. In particular, following recommendations from previous studies [20,21], novelty was examined from the perspective of social factors, or to be more precise, from support for the BPNs given by the physical education teacher. In this sense, this investigation attempts to fulfil the established third, fourth and fifth criteria in order to be considered as a new basic psychological need. The third criterion refers to the attempt to see the behavior of novelty as a mediator between social factors and its relationship with motivation. The fourth refers to work in synergy with the other basic psychological needs being a growing need, instead of a deficit need. Finally, the fifth refers to confirm novelty as a predictive factor of self-determined motivation [1]. With this aim in mind, two principal objectives were proposed. The first objective was to examine the psychometric properties from the Support for the Basic Psychological Needs in Physical Education questionnaire (SBPN4), which included support for novelty. The second objective attempted to test a mediation model where support for novelty would predict the BPNs and that these in turn would predict the intrinsic motivation of the students, including and examining the role played by novelty.

Firstly, and with respect to the first objective, the items with the greatest content validity were selected from those proposed by a group of experts, based on a selection of works on novelty up to the time of the study. Then, a confirmatory factor analysis was completed according to a simple structure of four factors with the possibility of correlation between them. The results showed both strong goodness-of-fit indices and high factorial loading. Due to the strong correlation indicated between the factors and following the recommendations of the original version [19], two different possible models were tested. In one of them, all elements were grouped within a single factor, while the other consisted of a model with a general second-order factor that explained the correlations between the four first-order factors. The model in which all elements were grouped within a single factor presented only acceptable goodness-of-fit indices. However, the model with the general second-order factor showed goodness-of-fit indices that were close to the original model, although with statistically significant differences. These results agree with the SDT [2], which clearly separates the distinct BPNs, from which specific strategies could be devised in order to satisfy each one [5,6,7,42]. Despite this, the SDT itself [2] specifies the relationship between them as belonging to a unique “basic psychological needs” construct, having been found to be strongly correlated with the other needs (including novelty) in most literature [20,21,22]. For this reason and on the basis of results obtained through factor analyses, use of the original model with four correlated factors is recommended. On the other hand, it is suggested to future investigators who use a questionnaire that, in relation to their objectives and results, they consider adding the global second-order factor “Support for BPNs” to the existing four factors in order to evaluate teacher support for the BPNs more globally. The comparison between means of the SBPN4 subscales showed that there were no statistically significant differences between boys and girls. Additionally, the participants perceived that their teachers provided more support for the need for novelty than for the need for autonomy, but less than they did for the needs for competence and relatedness. The remaining psychometric tests completed with SBPN4—such as invariance with respect to gender, internal consistency, temporal stability, etc.—showed good indices [35,36,37,38], thus indicating adequate validation of the questionnaire. 

Regarding the second objective, the proposed model [20,21] that evaluated the role of novelty from the perspective of social factors was tested. The hypothesis put forward was that support for novelty would predict satisfaction of the BPNs (including satisfaction of novelty), which, in turn, would predict intrinsic motivation. Therefore, a mediation model was proposed, as had been done in another recent investigation [23], but using support for novelty from the SBPN4 questionnaire as a dimension, instead of working environment, thus focusing purely on the Theory of Basic Psychological Needs. The results of this analysis have shown how the effect of support for novelty on intrinsic motivation was partially mediated by satisfaction of the needs for novelty, autonomy, and competence. The effect of satisfaction of the BPNs (including novelty) on more self-determined motivation has been widely demonstrated in previous studies [20,21,22], although showing inconsistent results with those concerning the need for relatedness in the present investigation [20]. However, until the present study, the role of support of the need for novelty had not been evaluated [26]. As expected, satisfaction of the need for novelty played a more significant mediating role than the satisfaction of other basic psychological needs. In turn, the effect of support for novelty was produced principally through satisfaction of novelty, which was reflected by a larger percentage of explained variance and a greater indirect effect when the satisfaction of novelty was considered as a mediator, compared with the mediating role of the satisfaction of the other BPNs. 

The results obtained may indicate that students are capable of perceiving when the teacher implements strategies for the satisfaction of novelty and also that these will have positive consequences for intrinsic motivation. Some of these strategies may be to introduce different content to the usual, such as alternative sports (e.g., tchoukball, kinball, goalball), trendy physical-expressive activities (e.g., pole dance, Zumba, bodycombat), or activities outside of the school environment (e.g., parkour or slackline in urban environments or beach sports in coastal environments). Another strategy with the aim of developing novelty could be the use of digital resources, such as mobile applications (e.g., Tik Tok, Munzee, HomeCourt, augmented reality applications), the use of activities that until now have been employed very little in PE classes (e.g., escape room or gamification), the use of different materials or of other innovative materials (e.g., fitball, suspension training equipment, recycled material, materials brought in from home by the students). In short, transformation of the teaching-learning process in order to create new possibilities and to cultivate curiosity will depend on the awareness, involvement, and creativity of teachers [22].

Despite the results obtained, it is necessary to take into account a number of considerations and to be cautious with regards to the inclusion of novelty as a fourth BPN. The present investigation has a number of limitations, such as the common method bias [43]. The means of support for the basic psychological needs were obtained only from the opinion of the students, meaning that the use of independent sources should perhaps be used on future occasions in order to evaluate support for novelty. Moreover, a transverse design, like the one used, is limited in terms of its capacity to establish the necessary temporal sequence for the study of mediational models. Therefore, in future investigations, the completion of longitudinal studies to examine the role of interventions focused on the satisfaction of novelty is recommended in order to more adequately test the mediating effects. Realization of these recommendations would help us to establish more fully and more exactly the effective strategies and methods for its satisfaction, given that the aforementioned proposals remain unexplored. Likewise, the creation of other observation instruments is proposed to help evaluate the application of these strategies; in order to clarify the differences between novelty and variety; as well as to help us understand when a stimulus ceases to be novel, along with its potential consequences. Perhaps both represent two facets of a more global construct [26,44]. The differentiation or not between surprise–variety–novelty is a line with great possibility of research. Finally, we propose that further study be completed to examine novelty using the entire proposal set out by the theory of self-determination [2] (social factors, satisfaction of BPNs, motivation and consequences) which would help to develop the established criteria [1].

## 5. Conclusions

The objective of the study was to test the behavior of the need for novelty as a possible fourth BPN in the self-determination theory [2], particularly from the perspective of social factors. As such, the psychometric properties of the Support for Basic Psychological Needs-4 (SBPN4) questionnaire, which included support for novelty as a fourth factor, were analyzed. The results obtained indicate that the SBPN-4 questionnaire is a valid and reliable tool. Second, the mediation model showed that support for novelty predicted the satisfaction of the BPNs (especially the satisfaction of novelty), and that these in turn, predicted intrinsic motivation. The results obtained provide evidence for the third, fourth and fifth criteria established for the inclusion of new BPNs in the SDT [1]. Despite the significance of these results, they must be taken with caution and further investigation is still required before we are able to consider novelty as the fourth pillar in the Theory of Basic Psychological Needs.

## Figures and Tables

**Table 1:** Estimates of factor loadings and factor covariances for the four correlated factors model of the SBPN4

Factor	Estimate (Standard.Error)	Standardized Estimate
	Factor Loadings	
Autonomy		
Item 1	1.00	0.66
Item 5	1.04(0.06)	0.70
Item 9	1.31(0.06)	0.84
Item 13	1.13(0.06)	0.77
Competence		
Item 2	1.00	0.82
Item 6	0.70(0.04)	0.60
Item 10	0.77(0.04)	0.75
Item 14	0.86(0.04)	0.80
Relatedness		
Item 3	1.00	0.82
Item 7	0.98(0.04)	0.81
Item 11	0.98(0.04)	0.77
Item 15	0.97(0.05)	0.73
Novelty		
Item 4	1.00	0.68
Item 8	1.23(0.05)	0.85
Item 12	1.12(0.05)	0.81
Item 17	1.17(0.06)	0.79
Factor Covariances		
Autonomy w/Competence	0.77(0.05)	0.86
Autonomy w/Relatedness	0.62(0.05)	0.79
Autonomy w/Novelty	0.66(0.05)	0.87
Competence w/Relatedness	0.86(0.07)	0.89
Competence w/Novelty	0.84(0.07)	0.89
Relatedness w/Novelty	0.66(0.06)	0.80

Note: All estimates were significant at *p* < 0.001.

**Table 2:** Confirmatory factor analysis fit statistics of the SBPN4.

Model	S-B χ2	df	CFI	TLI	RMSEA (90% CI)	SRMR
Independence (null) model	5289.28 *	120				
One-factor model	539.59 *	104	0.916	0.903	0.076 (0.071, 0.082)	0.043
Four-factor model	221.59 *	98	0.976	0.971	0.042 (0.035, 0.048)	0.027
Second-order model	238.47 *	100	0.973	0.968	0.044 (0.038, 0.050)	0.030

Note: * *p* < 0.001. S-B χ2: Satorra–Bentler chi-square. Df: degrees of freedom. CFI: Comparative fit index. RMSEA (90% CI): Root mean square error of approximation (90% confidence interval). SRMR: Standardized root mean square.

**Table 3:** Measurement invariance tests of the SBPN4 four-factor model across gender.

Model (Invariance Level)	Overall Fit						Model Comparison	Comparative Fit				
	S-B χ2	df	CFI	TLI	RMSEA	SRMR		ΔS-B χ2	Δdf	*p*	ΔCFI	ΔRMSEA
1. Configural	342.46 *	196	0.972	0.966	0.045	0.033						
2. Metric	361.81 *	208	0.970	0.966	0.045	0.039	2 vs. 1	18.83	12	0.093	−0.002	0.000
3. Scalar	385.21 *	220	0.968	0.965	0.046	0.040	3 vs. 2	24.35	12	0.018	−0.002	0.001
4. Uniqueness	399.71 *	236	0.969	0.968	0.044	0.040	4 vs. 3	15.37	16	0.498	0.003	−0.002

Note: * *p* < 0.001. S-B χ2: Satorra–Bentler chi-square. df degrees of freedom. CFI: Comparative fit index. RMSEA (90% CI): Root mean square error of approximation (90% confidence interval). SRMR: Standardized root mean square ΔS-B χ2: Increment in Satorra–Bentler chi-square. Δdf: Increment in degrees of freedom. *p*: *p*-value. ΔCFI: Increment in comparative fit index. ΔRMSEA: Increment in root mean square error of approximation.

**Table 4:** Reliability, temporal stability, descriptive statistics, and correlations of study variables

Variables	α	ω	Range	M	SD	1	2	3	4	5	6	7	8	9	ICC
Novelty Support	0.86	0.86	1–5	3.51	1.08		0.73	0.76	0.69	0.67	0.57	0.35	0.25	0.60	0.92
Autonomy Support	0.83	0.83	1–5	3.00	1.04			0.73	0.68	0.61	0.60	0.35	0.25	0.51	0.90
Competence Support	0.83	0.83	1–5	3.91	0.96				0.77	0.61	0.58	0.42	0.28	0.63	0.93
Relatedness Support	0.86	0.86	1–5	3.97	0.97					0.51	0.51	0.37	0.37	0.53	0.90
Novelty Satisfaction	0.86	0.86	1–5	3.47	0.92						0.68	0.48	0.39	0.66	
Autonomy Satisfaction	0.79	0.79	1–5	2.97	0.93							0.57	0.34	0.62	
Competence Satisfaction	0.78	0.79	1–5	3.72	0.89								0.52	0.58	
Relatedness Satisfaction	0.81	0.82	1–5	4.18	0.87									0.40	
Intrinsic Motivation	0.85	0.85	1–7	5.31	1.40										

Note: α: Cronbach Alpha. ω: McDonald omega. M: Mean. SD: Standard deviation. ICC: Intraclass correlation coefficient. All correlations were significant at *p*< 0.001.

## Appendix A. Support for Basic Psychological Needs-4 (SBPN4)

**Table A1:** This questionnaire was validated in Spanish.

In Physical Education Classes, Our Teacher…	En las Clases de Educación Física, Nuestro/a Profesor/a…
1. Often asks us about the activities we want to do	1. Nos pregunta a menudo sobre nuestras preferencias respecto a las actividades a realizar
2. Encourages us to trust in our ability to complete the tasks well	2. Nos anima a que confiemos en nuestra capacidad para hacer bien las tareas
3. Always fosters good relationships between classmates	3. Fomenta en todo momento las buenas relaciones entre los compañeros/as
4. Does different things to what we are used to	4. Hace cosas distintas respecto a lo que estamos acostumbrados/as
5. Tries to give us freedom when we are completing the activities	5. Trata de que tengamos libertad a la hora de realizar las actividades
6. Proposes activities that are tailored to our level	6. Nos propone actividades ajustadas a nuestro nivel
7. Promotes a positive environment among classmates	7. Favorece el buen ambiente entre los compañeros/as de clase
8. Frequently suggests new activities	8. Nos propone actividades novedosas con frecuencia
9. Considers our own opinions on how to run the classes	9. Tiene en cuenta nuestra opinión en el desarrollo de las clases
10. Always tries to make sure we achieve the activities’ objectives	10. Siempre intenta que consigamos los objetivos que se plantean en las actividades
11. Makes all the students feel involved	11. Promueve que todos los alumnos/as nos sintamos integrados
12. Helps us to discover new things	12. Nos ayuda a descubrir cosas nuevas
13. Lets us make our own decisions during the tasks	13. Nos deja tomar decisiones durante el desarrollo de las tareas
14. Encourages learning and improvement during classes	14. Fomenta el aprendizaje y la mejora de los contenidos de la asignatura
15. Helps us to resolve conflicts amicably	15. Nos ayuda a resolver los conflictos amistosamente
16. Frequently inspires our curiosity	16. Con frecuencia despierta nuestra curiosidad
